# Role of SNARE Proteins in the Insertion of KCa3.1 in the Plasma Membrane of a Polarized Epithelium

**DOI:** 10.3389/fphys.2022.905834

**Published:** 2022-06-27

**Authors:** Rachel E. Farquhar, Tanya T. Cheung, Matthew J. E. Logue, Fiona J. McDonald, Daniel C. Devor, Kirk L. Hamilton

**Affiliations:** ^1^ Department of Physiology, School of Biomedical Sciences, University of Otago, Dunedin, New Zealand; ^2^ Department of Cell Biology, University of Pittsburgh, School of Medicine, Pittsburgh, PA, United States

**Keywords:** K^+^ channels, VAMP3, SNAP-23, syntaxin-4, EBIO, clotrimazole

## Abstract

Targeting proteins to a specific membrane is crucial for proper epithelial cell function. KCa3.1, a calcium-activated, intermediate-conductance potassium channel, is targeted to the basolateral membrane (BLM) in epithelial cells. Surprisingly, the mechanism of KCa3.1 membrane targeting is poorly understood. We previously reported that targeting of KCa3.1 to the BLM of epithelial cells is Myosin-Vc-, Rab1-and Rab8-dependent. Here, we examine the role of the SNARE proteins VAMP3, SNAP-23 and syntaxin 4 (STX-4) in the targeting of KCa3.1 to the BLM of Fischer rat thyroid (FRT) epithelial cells. We carried out immunoblot, siRNA and Ussing chamber experiments on FRT cells, stably expressing KCa3.1-BLAP/Bir-A-KDEL, grown as high-resistance monolayers. siRNA-mediated knockdown of VAMP3 reduced BLM expression of KCa3.1 by 57 ± 5% (*p* ≤ 0.05, *n* = 5). Measurements of BLM-localized KCa3.1 currents, in Ussing chambers, demonstrated knockdown of VAMP3 reduced KCa3.1 current by 70 ± 4% (*p* ≤ 0.05, *n* = 5). Similarly, siRNA knockdown of SNAP-23 reduced the expression of KCa3.1 at the BLM by 56 ± 7% (*p* ≤ 0.01, *n* = 6) and reduced KCa3.1 current by 80 ± 11% (*p* ≤ 0.05, *n* = 6). Also, knockdown of STX-4 lowered the BLM expression of KCa3.1 by 54 ± 6% (*p* ≤ 0.05, *n* = 5) and reduced KCa3.1 current by 78 ± 11% (*p* ≤ 0.05, *n* = 5). Finally, co-immunoprecipitation experiments demonstrated associations between KCa3.1, VAMP3, SNAP-23 and STX-4. These data indicate that VAMP3, SNAP-23 and STX-4 are critical for the targeting KCa3.1 to BLM of polarized epithelial cells.

## Introduction

KCa3.1 (Syn, IK1, SK4) is an intermediate-conductance Ca^2+^-activated K^+^ channel that plays crucial roles in maintaining the electrochemical driving force for Ca^2+^-mediated transepithelial Cl^−^ secretion across polarized epithelia (Flores et al., 2007; Balut et al., 2012; Devor et al., 2016; 2020). KCa3.1 has been well established as a channel that resides in the BLM of epithelial cells (Devor et al., 1996; 1999; Rufo et al., 1996; Hamilton et al., 1999; Hamilton and Kiessling 2006), and non-epithelial tissues (Neylon et al., 1994, 2004; Rapetti-Mauss et al., 2015; Turner et al., 2015; Storck et al., 2017). KCa3.1 is critical in a wide range of physiological functions in both epithelia and non-epithelial tissues. With such a wide distribution, KCa3.1 has been touted as a potential target in a range of diseases (Devor et al., 1996; Damkjaer et al., 2012; Bradding and Wulff, 2009; Köhler et al., 2010; Wulff and Köhler, 2013; Brown et al., 2020; Devor et al., 2020). Indeed, altered numbers of KCa3.1 channels expressed at the cell plasma membrane results in pathophysiological conditions such as ulcerative colitis (UC) and polycystic kidney disease (PKD). In UC, reduced expression of KCa3.1 in colonic cells resulted in decreased driving forces for ion, solute and water absorption within the colon resulting in diarrhea contribuing to UC (Albaqumi et al., 2008). In PKD, the number of KCa3.1 is increased resulting in a greater driving force for Cl^−^ secretion increasing water secretion crucial in fluid accumulation in cysts associated with PKD (Al-Hazza et al., 2012).

The physiological response of KCa3.1 at the BLM of epithelial cells is determined by both the number of channels residing in the membrane and the open probability of the channels capable of responding to an agonist. We (Gerlach et al., 2000; 2001; Jones et al., 2004; 2005; 2007; Syme et al., 2003; Gao et al., 2008) and others (Klein et al., 2007; Garneau et al., 2009) have examined the opening properties of KCa3.1 and have identified motifs which affect assembly and trafficking of the channel to the plasma membrane. However, the resident population of channels at the BLM, at any given point in time, is dependent upon the rates of anterograde and retrograde trafficking of the channels.

Our understanding of the anterograde trafficking of KCa3.1 in epithelial cells has advanced over the past years. Bertuccio et al. (2014) reported that trafficking and maintenance of KCa3.1 at the BLM is dependent on Rab-1 and Rab-8 while being independent of recycling endosomes, and the AP-1 adaptor protein, µ1B. Additionally, we reported that trafficking of KCa3.1 to the BLM is a cytoskeletal- and myosin-Vc-dependent process (Farquhar et al., 2017). However, the final molecular process (es) of tethering, docking and insertion of KCa3.1-containing vesicles at the BLM remains to be elucidated.

Anterograde trafficking of proteins to a specific cellular membrane is a complex process (Carmosino et al., 2010; Sauvola and Littleton, 2021; Koike and Jahn, 2022). The Soluble *N*-ethylmaleimide-sensitive factor (SNF) Attachment protein REceptors, (SNAREs), are a family of proteins that assist with the docking of vesicles carrying cargo, such as ion channels, to a specific target membrane (Low et al., 1996; Condliffe et al., 2003, 2004; Fields et al., 2007; Pocard et al., 2007). In particular, three SNAREs, Vesicle-associated membrane protein 3, v-SNARE (VAMP3); SNAP-23, Soluble NSF Attachment Protein (SNAP23); and syntaxin 4 target (t)-SNARE (STX-4) play important roles in targeting proteins to the basolateral membrane of epithelial cells (Gaisano et al., 1996, 1997; Low et al., 1996, 1998; Leung et al., 1998; Li et al., 2002; Fields et al., 2007). Generally, the t-SNAREs localize to a specific membrane (e.g., basolateral membrane) where these proteins mediate the fusion of incoming vesicles in the trafficking pathways to a particular membrane via a v-SNARE (Torres et al., 2011). Recently, Bertuccio et al. (2018) reported that the plasma membrane insertion of KCa2.3, another member of the KCNN family as KCa3.1, was dependent upon SNAP-23 and STX-4.

In this study, we investigated the role of these SNARE proteins in the BLM trafficking of KCa3.1-containing vesicles in a polarized epithelium. Here, we provide the first evidence that targeting of KCa3.1 to the BLM is a VAMP3-, SNAP-23-, and STX-4 -dependent process. Indeed, this is the first report of the participation of VAMP-3 in the trafficking of any member of the KCNN gene family. Knockdown of all three SNARE proteins reduced expression of KCa3.1 at the BLM of epithelial cells and reduced the membrane functional expression of KCa3.1. Additionally, with co-immunoprecipitation (Co-IP) experiments, we identified protein associations between KCa3.1 and VAMP3, SNAP-23 and STX-4. In total, our results suggest insertion of KCa3.1 into the BLM of epithelial cells is critically dependent on VAMP3, SNAP-23 and STX-4. Therefore, disruption or reduction of any of these SNARE proteins would result in reduced functional activity of KCa3.1.

## Materials and Methods

### Molecular Biology

The biotin ligase acceptor peptide (BLAP) sequence (GLNDIFEAQKIEWHE) was inserted into the extracellular loop of KCa3.1 between the transmembrane domains S3 and S4 as previously described (Balut et al., 2010a; Gao et al., 2010; Farqhuar et al., 2017; Lee et al., 2017). KCa3.1-BLAP and BirA (biotin ligase) with an endoplasmic reticulum (ER) retention sequence, KDEL (kindly provided by Dr. Alice Ting, Massachusetts Institute of Technology, Cambridge, MA, Chen et al., 2005; Howarth and Ting, 2008), were subcloned into a bicistronic plasmid, pBudCE4.1 (Invitrogen, Carlsbad, CA, United States) behind the EF-1α and CMV promoters, respectively (Balut et al., 2010a; 2010b; Gao et al., 2010; Balut et al., 2011).

### Cell Culture and Fischer Thyroid Rat Cell Line Stably Expressing KCa3.1-Biotin Ligase Acceptor Peptide and BirA-KDEL

Fischer rat thyroid (FRT) cells were used as a cell model of a polarized epithelium (Zurzolo et al., 1991). A stable FRT cell line was generated by transfecting in the bicistronic plasmid expressing both KCa3.1-BLAP and BirA-KDEL into FRT cells using Lipofectamine 2000 (Invitrogen) following the manufacturer’s instructions and selecting a stable cell line using zeocin (850 μg/ml). We have previously demonstrated that insertion of the BLAP sequence into KCa3.1 did not affect the Ca^2+^ sensitivity, activation by DCEBIO, or the inhibition by clotrimazole (Gao et al., 2010). Both untransfected FRT and the KCa3.1-BLAP + BirA-KDEL FRT cell lines were cultured in Nutrient Mixture F-12 (Ham’s F-12; Sigma Aldrich, St. Louis, MO) at a pH of 7.4, followed by the addition of 10% fetal bovine serum and 1% penicillin-streptomycin (Life Technologies, NZ). Cells were grown in 25 cm^2^ flasks (NUNC™, ThermoFisher Scientific, Waltham, MA, United States) and incubated in a humidified 5% CO_2_, 95% air incubator at 37°C. For all experiments, untransfected and stably expressing cells were grown (72 h) on Transwell™ (#COR3406) or Snapwell™ (#COR801) support filters (Corning, NY, United States) to establish a polarized epithelium and allow access to both apical and basolateral sides of the epithelium. All experiments were conducted with cells from passage numbers 11–27 for this study. The University of Otago Institutional Biological Safety Committee approved all experimental protocols and the molecular biological techniques used in this study.

### Biotinylation and Streptavidin Labeling of KCa3.1-Biotin Ligase Acceptor Peptide

Within the stable KCa3.1-BLAP BirA-KDEL cell line, the BirA-KDEL is retained within the ER. Therefore, after assembly, KCa3.1-BLAP is biotinylated in the ER prior to being trafficked out to the plasma membrane. Streptavidin labeling of surface KCa3.1-BLAP was performed as previously described (Balut et al., 2010a; Gao et al., 2010; Bertuccio et al., 2014; Farquhar et al., 2017; Lee et al., 2017). Briefly, upon reaching confluence (72 h after cell seeding), cells were taken out of the incubator for labeling; all procedures and solutions were maintained at 4°C to prevent channel internalization. Cells were first washed with 2 ml of 4°C phosphate buffered saline (PBS) with 1% bovine serum albumin (BSA) on both apical and basolateral sides of the permeable support filter to eliminate residual media. Since streptavidin is cell impermeable, the cells were labeled by applying streptavidin (10 μg/ml of streptavidin in PBS with 1% BSA) on the desired side (apical or basolateral, or both) for 30 min at 4°C. After labeling, cells were washed three times with PBS with 1% BSA and three times containing PBS to eliminate residual streptavidin and incubated for various periods of time at 37°C, as indicated in the text.

### siRNA Reverse Transfection of Fischer Rat Thyroid Cells

FRT-KCa3.1-BLAP cells were reverse transfected (20 pmol) with either VAMP3-siRNA (Millennium Science, NZ, SMARTpool siGenome VAMP3 siRNA, Cat. No. M-088738–01), SNAP-23-siRNA (Santa Cruz Biotechnology Cat. No. SANTSC-72219) or STX4-siRNA (Life Technologies, NZ, Cat. No. 1330003) or control siRNA (Stealth™ RNAi Negative control (Invitrogen, Cat. No. 12935300) the sequence is not disclosed by Invitrogen) for 48 h using Lipofectamine 2000™ (Invitrogen) following the manufacture’s protocol. FRT-KCa3.1-BLAP cells were seeded at a density of 7.5 × 10^4^ cells onto Transwell™ and Snapwell™ permeable support filters (Corning, NY, United States) and cultured to form a confluent epithelial monolayer in a humidified 5% CO_2_/95% air incubator at 37°C.

### Immunoblot Experiments

**FIGURE 1:**
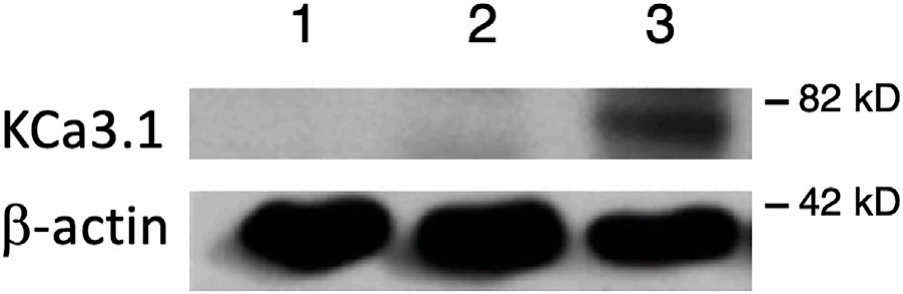
Membrane location of KCa3.1-BLAP stably expressed in FRT cells. The sidedness experiments confirm the expression pattern of KCa3.1 on the basolateral membrane of FRT cells. Untransfected FRT cells or FRT-KCa3.1-BLAP cells were grown on Transwell™ filter inserts to form a monolayer. Untransfected cells were labeled with streptavidin on both sides of the filter; while FRT-KCa3.1-BLAP cells were labeled either on the apical side or the basolateral side of the filter insert. A band was only observed in the sample that was labeled basolaterally near the 82 kD mark. Lane 1: untransfected FRT, Lane 2: KCa3.1-BLAP- + Apical streptavidin labeling, and Lane 3: KCa3.1-BLAP + Basolateral streptavidin labeling. Each lane was loaded with 30 µg of protein and β-actin was used as a loading control (*n* = 5).

Immunoblot (IB) experiments were performed as described previously (Jones et al., 2004, 2007; Balut et al., 2010a, 2010b; Gao et al., 2010; Bertuccio et al., 2014; Farquhar et al., 2017.; Lee et al., 2017). Cells were lysed and the protein was harvested and protein concentrations were determined by the bicinchoninic acid protein assay technique (Walker, 1994). 30 μg of protein was run per lane with a protein standard (BenchMark™, Invitrogen, Cat. No. 10748–010) added to a different lane and separated on an 8% SDS-PAGE gel, gels were run on a Hoefer Mighty Small II system (Cat. No. 80–6,149-35, Amersham Biosciences Corp. Piscataway, NJ, United States) at 150 V for 90 min or until the dye front reached the bottom of the gel. After which, proteins were transferred onto a polyvinyl-diflouride membrane (Sigma, St. Louis, MO, United States) using the Trans-Blot^®^ Turbo™ Transfer Starter System (Model 1,704,155, Bio-Rad, Hercules, CA, United States) in transfer buffer (25 mM Tris, 190 mM glycine and 20% methanol) at 25 V for 30 min. Membranes were blocked overnight at 4°C in a TBS-T blocking solution (5% milk powder, 50 mM Tris, 150 mM NaCl, 0.1% Tween 20). Membranes were then incubated in 1° antibody for 1 h at room temperature for the detection for either KCa3.1, a SNARE protein, or GAPDH (used as a loading control), although, β-actin was used as the loading control for the initial KCa3.1-BLAP sidedness expression experiments (Figure 1). Membranes were then washed extensively in TBS-T (0.1% Tween 20) and incubated in the appropriate 2° antibody for 1 h at room temperature. Membranes were then washed again in TBS-T (0.1% Tween 20) and detection was performed using West Pico Chemiluminescent Substrate (Roche Diagnostics, Indianapolis, IN, United States). Immunoblot band densities for KCa3.1, VAMP3, SNAP-23, and STX-4 were normalized to the GAPDH or β-actin loading controls. All immunoblot data were quantified using ImageJ (NIH, vers. 1.51, Bethesda, MD, United States).

### Antibodies

Antibody dilutions were as follows streptavidin antibody (rabbit 1^o^ Ab 1:1,000, Genescript pAb Cat. No. A00621, 2^o^ donkey anti-rabbit 1:10,000, Amersham Cat. No. NA934, Sigma-Aldrich, New Zealand (NZ)); VAMP3 antibody (rabbit 1^o^ Ab 1:1,000, Sapphire Bioscience LS- Cat. No. C144617, NZ, 2^o^ donkey anti-rabbit 1:10,000); SNAP-23 (mouse 1^o^ Ab 1:1,000, Santa Cruz Biotechnology (A5) Cat. No. SC-166244 Dallas, TX, 2^o^ goat anti-mouse 1:2000 Sigma-Aldrich Cat. No. A4416, NZ); STX-4 (rabbit 1^o^ Ab 1:1,000, Merck Cat. No. 574788, Germany, 2^o^ donkey anti-rabbit 1:10,000); GAPDH (rabbit 1^o^ Ab 1:1,000, Sigma-Aldrich Cat. No. G9545, NZ); and β-actin IgG antibody (mouse 1:10,000, Sigma-Aldrich Cat. No. A5441, NZ).

### Co-Immunoprecipitation Experiments

Co-immunoprecipitation (Co-IP) experiments were used to investigate specific protein–protein associations between the SNARE proteins and KCa3.1. One protein was used as a ‘bait’ protein for detecting a second ‘target’ protein. Firstly, FRT–KCa3.1 cells were cultured for 72 h on Transwell^®^ filters, as described above, before being labelled with streptavidin and lysed. The protein concentration of the lysates was determined using the DC assay (BioRad, United States).

A sample of 30 µg of protein was retained for whole cell lysate/input. Equal amounts of the remaining lysate were incubated with 2 µL of antibody and incubated at 4°C for 2 h with gentle rotation. Next, 50 µL of PBS-equilibrated Protein G Sepharose^®^ beads (Sigma-Aldrich, Cat. No. P3296) was added to each microcentrifuge tube, before being incubated for a further 1 h at 4°C with gentle rotation. After incubation, the beads were pelleted by centrifuging at 400 × *g* for 1 min at 4°C, the supernatant was discarded, and the beads were washed with 1 ml of TBS with 1% (v/v) Triton™ X-100. This wash step was repeated a further 5 times. After the final wash step, 5 × sample buffer (150 mM Tris-HCl, 5% SDS, 26% glycerol, 10% *β*-mercaptoethanol, 0.08% bromophenol blue, pH 6.8) was added to a final volume of 50 µL. Samples were then denatured by heating at 95°C on a dry heat block for 5 min. Samples were analyzed by immunoblotting, as previously described. Goat anti-rabbit IgG antibody was used for the negative control in VAMP3/SNAP-23/STX-4, and KCa3.1 Co-IPs.

### Stripping Protocol to Re-Probe for Another Protein

For the Co-IP experiments, some PVDF membranes were re-probed with a second antibody to detect other possible protein associations with the bait protein. Prior to immunodetection with the new antibody, the membrane was stripped by incubating in stripping buffer (25 mM glycine, 0.1% Tween-20, 1% w/v SDS, pH 2.0) for 10 min at room temperature. Following incubation, the membrane was washed with TBS-T 3 times for 10 min each, before being re-blocked and re-probed.

### Ussing Chamber Experiments

Ussing chamber experiments were conducted to examine the effect of knockdown of SNARE proteins on the functional expression of KCa3.1 at the BLM, as measured as K^+^ currents (I_K_). I_K_ was measured by a VCC MC Ussing chamber system that consisted of an Easymount chamber system and an 8-channel voltage/current clamp unit (Physiologic Instruments, San Diego, CA, United States) as previously described (Farquhar et al., 2017; Lee et al., 2017). FRT cells were grown on Snapwell™ filters for 5 days prior to an experiment and transfection of control or siRNA occurred 48 h prior to the experiments (details are stated in the text). Once a filter was mounted into a Ussing chamber, the apical (mucosal) surface of the monolayer was bathed in a solution containing (in mM) 145 potassium gluconate, 10 HEPES, 1 MgCl, 4 CaCl_2_ and 10 glucose (pH 7.4) and the basolateral (serosal) surface was bathed in a solution containing (in mM) 140 sodium gluconate, 5 potassium gluconate, 10 HEPES, 1 MgCl, 4 CaCl_2_ and 10 glucose (pH of 7.4). All solutions were maintained at 37°C. The CaCl_2_ was increased from the normal 1.2–4 mM to compensate for the Ca^2+^-buffering capacity of the gluconate anion (Durham, 1983). These Ringer’s solutions facilitated a K^+^ gradient across the monolayer and promoted transepithelial K^+^ transport. Prior to mounting a filter in a chamber, the Ussing chamber was zeroed to remove any offsets (Clarke, 2009). The FRT monolayer was considered sufficient when it exhibited transepithelial resistance R ≥ 500 Ω cm^2^ therefore, experiments with low R were excluded.

To assess the effect of knockdown of SNAREs on the functional expression of KCa3.1 at the BLM, I_K_ via KCa3.1 was measured which consisted of stimulation of KCa3.1 with the addition of 1-EBIO consisted (mucosal (m) and serosal (s) sides of the filter), a KCa3.1 specific activator (Devor et al., 1996), and inhibiting the KCa3.1-stimulated current by the addition of clotrimazole (10 μM, m and s) (Devor et al., 1997). Therefore, using the combination of 1-EBIO and clotrimazole allowed the determination of the effect of SNAREs on the targeting of KCa3.1 to the BLM as measured by I_K_. Stably-transfected FRT cells transfected with control siRNA served as controls. We measured 1-EBIO stimulated–CLT-inhibited current as the difference of the plateau phase current stimulated by 1-EBIO compared with the current reduced by CLT. We have previously reported that FRT cells not transfected with KCa3.1-BLAP and BirA-KDEL do not exhibit 1-EBIO stimulated- or clotrimazole-sensitive current (Farquhar et al., 2017; Lee et al., 2017).

### Chemicals

All chemicals were purchased from Sigma-Aldrich, unless otherwise stated. The vehicle for 1-EBIO and clotrimazole was ethanol.

### Statistical Analyses

Recorded Ussing traces were analyzed using Microsoft Excel (2010) and GraphPad Prism 5 (GraphPad Software, Inc., La Jolla, CA). A nonparametric Kruskal–Wallis with a Dunn’s post-test was used to compare traces of 1-EBIO stimulated KCa3.1 currents of Ussing chamber data which were normalized to FRT-KCa3.1-BLAP controls. To compare the normalized values of the immunoblot band intensities, statistical analysis was performed using the nonparametric Kruskal–Wallis test followed by a Dunn’s post-test. A one-way ANOVA followed by a Bonferroni post-test was used to compare trace peaks of KCa3.1 current in FRT-KCa3.1-BLAP, FRT-KCa3.1-BLAP + SC-siRNA and FRT-KCa3.1-BLAP + SNARE-siRNA cell lines. All experiments were repeated from different passages of cells at least three times to ensure the fidelity of the results. All data are presented as mean ± SEM (standard error of the mean) and *p* ≤ 0.05 was considered statistically significant.

## Results

### Membrane Location of KCa3.1 in the Fischer Rat Thyroid KCa3.1-Biotin Ligase Acceptor Peptide Cell Line

It has been reported that there can be reduced functional expression of transport proteins in stable cell lines at high passage numbers (Yu et al., 1997). Therefore, initially, we confirmed the membrane expression of the KCa3.1-BLAP channel of our stably transfected FRT cell line. KCa3.1-BLAP cells were cultured on Transwell™ filters and labelled with streptavidin at either the apical or basolateral membrane. This was followed by immunoblot experiments using streptavidin and β-actin antibodies as described in the Materials and Methods. Untransfected FRT cells served as a negative control. As seen in Figure 1, KCa3.1 was highly expressed in the BLM (Lane 3) of the polarized epithelial cells (n = 5). These results confirm previous findings that KCa3.1 is targeted to the BLM in our stably-transfected FRT KCa3.1-BLAP cell line (Bertuccio et al., 2014; Farquhar et al., 2017; Lee et al., 2017).

### Role of VAMP3 in the Targeting of KCa3.1-Containing Vesicles to the Basolateral Membrane

**FIGURE 2:**
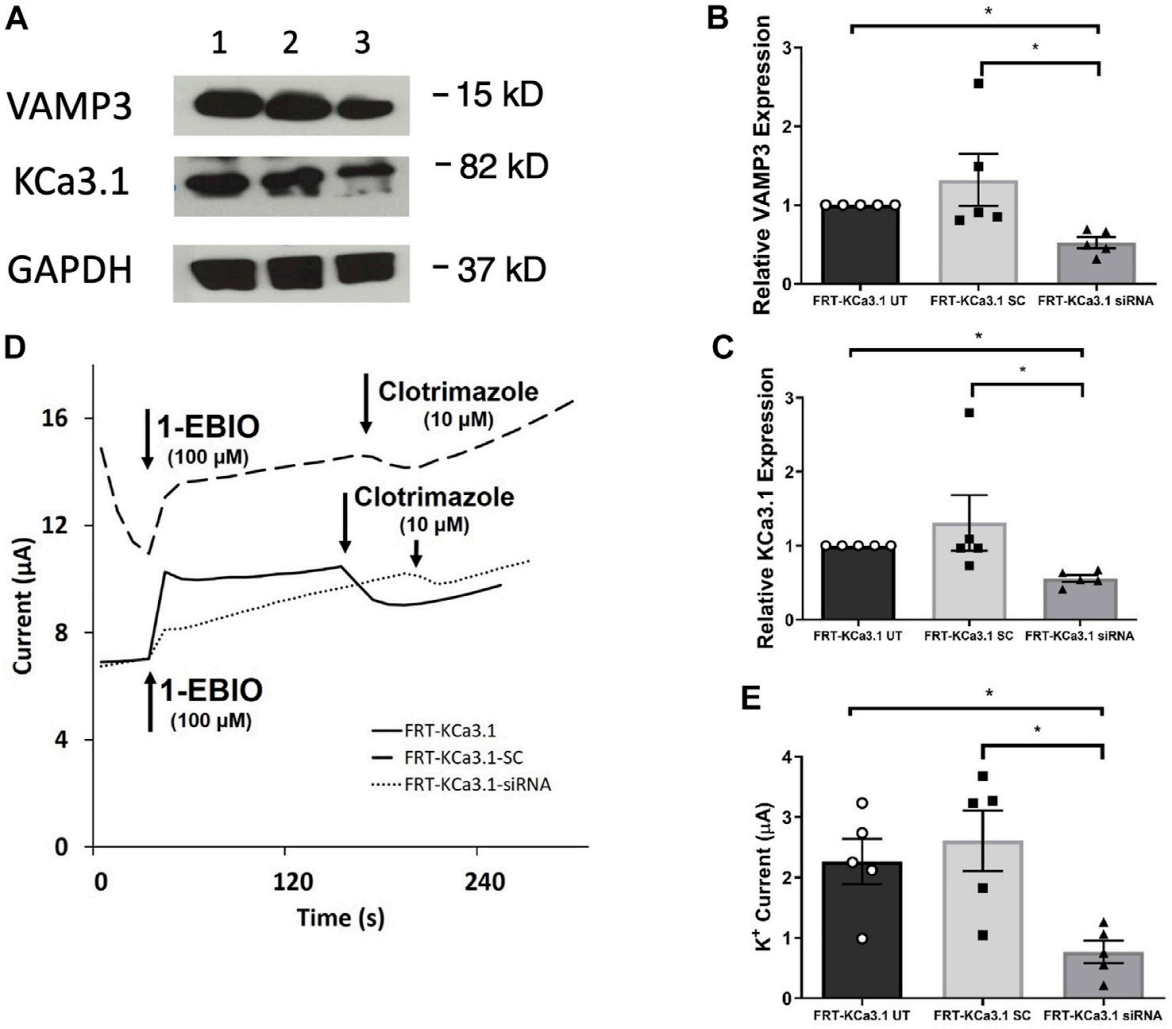
The membrane expression and function of KCa3.1 in response to the siRNA knockdown of VAMP3. FRT-KCa3.1-BLAP cells were reverse transfected (20 pM, 48 h) with a scrambled siRNA (FRT-KCa3.1-SC) or siRNA to knock down VAMP3 (FRT-KCa3.1-siRNA). **(A)** Proteins from control FRT-KCa3.1 UT (untransfected with any siRNA, UT), FRT-KCa3.1-SC, and FRT-KCa3.1-siRNA cells were run by immunoblot (8% gel). Lanes were loaded with 30 μg of protein. VAMP3 and KCa3.1 were detected in FRT-KCa3.1 cells. Lane 1: FRT-KCa3.1 UT, Lane 2: FRT-KCa3.1-SC, and Lane 3: FRT-KCa3.1-siRNA. GAPDH was used as a loading control. **(B)** VAMP3 expression in FRT-KCa3.1-siRNA cells was reduced by 61 ± 7% (*n* = 5, *p ≤ 0.05) with respect to FRT-KCa3.1-SC cells. **(C)** In the presence of knockdown of VAMP3, the KCa3.1 membrane expression in FRT-KCa3.1-siRNA cells was reduced by 57 ± 5% (*n* = 5, *p ≤ 0.05) with respect to FRT-KCa3.1-SC cells. **(D)** Ussing chamber experiment of FRT-KCa3.1 cells with VAMP3 knock down. Representative traces of control FRT-KCa3.1 cells (solid line), FRT-KCa3.1-SC control cells (dashed line) and FRT-KCa3.1-siRNA cells (dotted line) are shown. 1-EBIO promoted a significantly reduced K^+^ current in the FRT-KCa3.1-siRNA cells compared to control FRT-KCa3.1 cells or the FRT-KCa3.1-SC control cells. This current was abated by clotrimazole (10 µM). **(E)** Bar graph of K^+^ current of control FRT-KCa3.1 UT, FRT-KCa3.1-SC and FRT-KCa3.1-siRNA cells treated with 1-EBIO. FRT-KCa3.1-siRNA cells demonstrated a significantly reduced current by 70 ± 9% (*n* = 5, *p ≤ 0.05) compared to control FRT-KCa3.1-SC cells.

Fields et al. (2007) reported that VAMP3 was localized to the basolateral membrane of Madin-Darby canine kidney (MDCK) cells and that this SNARE protein played an integral part of the molecular machinery for delivering cargo to the BLM of polarized epithelial cells. Therefore, we hypothesized that reduced cellular VAMP3 would decrease trafficking of KCa3.1 to the BLM. In order to test this, we targeted VAMP3 using a siRNA approach. Therefore, FRT-KCa3.1-BLAP cells were transfected with either a universal negative control scrambled siRNA (FRT-KCa3.1-SC) or with a VAMP3-specific siRNA (FRT-KCa3.1-siRNA) and grown as stated in the Methods. FRT-KCa3.1-BLAP cells un-transfected with any siRNA served as controls. After 48 h post-transfection, cells were labelled with basolaterally applied streptavidin and prepared for immunoblot. The immunoblot data confirmed that the siRNA reduced cellular VAMP3 by 61 ± 7% (Figure 2A, upper panel, lanes 2 and 3) compared with the FRT-KCa3.1-SC cells (Figure 2B, dark gray versus light gray bar, *p* ≤ 0.05, n = 5). Similarly, in the presence of reduced cellular VAMP3, basolateral membrane expression of KCa3.1 was decreased by 57 ± 5% (Figure 2A, middle panel, lanes 2 and 3) compared to the FRT-KCa3.1-SC cells (Figure 2C, dark gray versus light gray bar, *p* ≤ 0.05, *n* = 5).

Next, we predicted that the functional expression of KCa3.1 would be reduced in the presence of VAMP3 siRNA due to reduced KCa3.1 at the BLM as seen in Figure 2C. Indeed, Ussing chamber experiments corroborated our immunoblot results. As seen in Figures 2D,E, knockdown of VAMP3 reduced the K^+^ current of the FRT-KCa3.1-siRNA cells (Figure 2D, dotted line, Figure 2E dark gray bar) by 70 ± 4% when compared with control FRT-KCa3.1-SC cells (Figure 2D, dash line, Figure 2E, light gray bar) (*p* ≤ 0.05, *n* = 5). These data suggest VAMP3 is required for delivery of KCa3.1 to the BLM in a polarized epithelia.

### Role of SNAP-23 in the Targeting of KCa3.1-Containing Vesicles to the Basolateral Membrane

**FIGURE 3:**
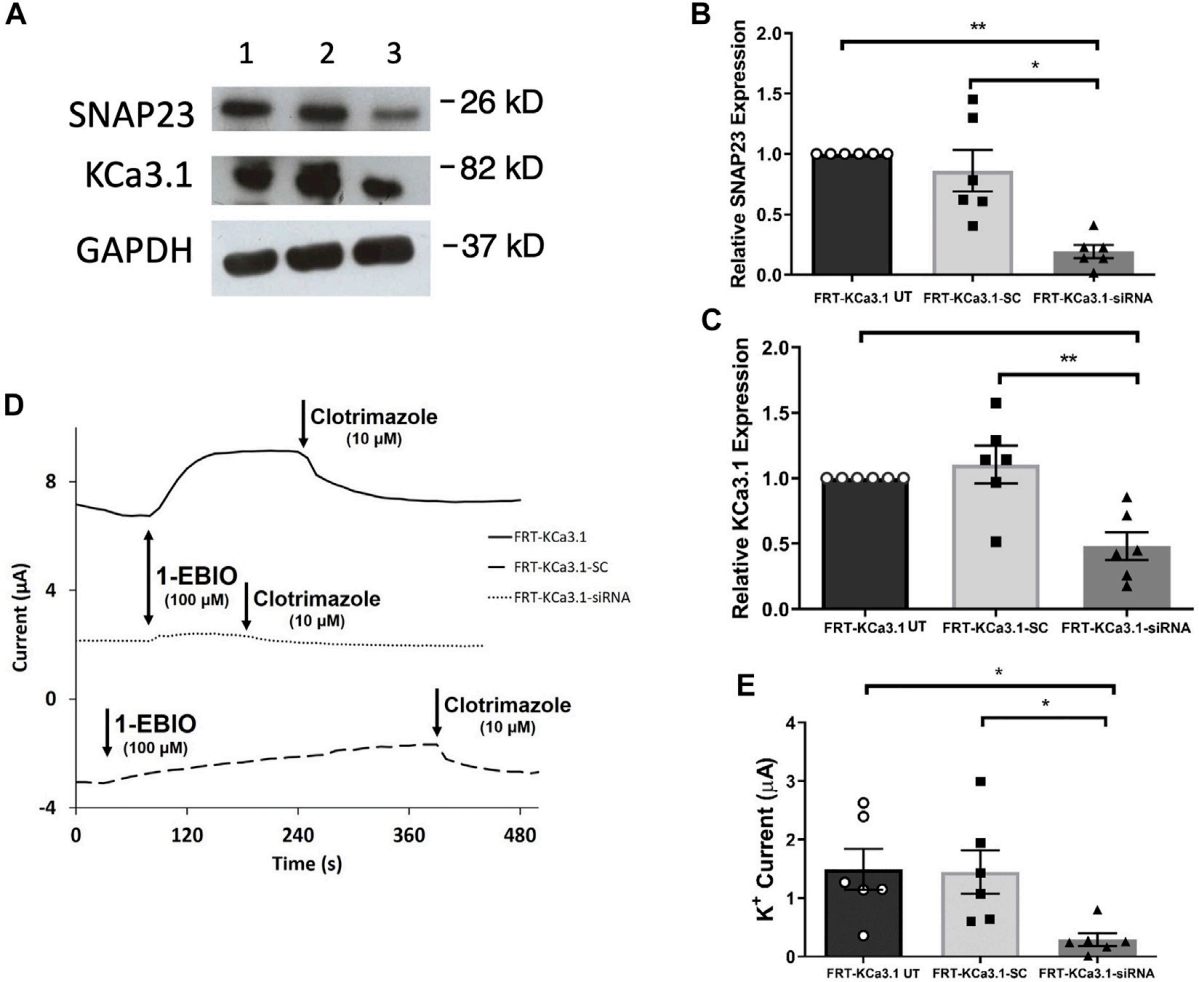
The membrane expression and function of KCa3.1 in response to the siRNA knockdown of SNAP-23. FRT-KCa3.1-BLAP cells were reverse transfected (20 pM, 48 h) with a scrambled siRNA (FRT-KCa3.1-SC) or siRNA to knock down SNAP-23 (FRT-KCa3.1-siRNA). **(A)** FRT-KCa3.1 UT (untransfected with any siRNA, UT), FRT-KCa3.1-SC, and FRT-KCa3.1-siRNA cells were run by immunoblot (8% gel). Lanes were loaded with 30 μg of protein. SNAP-23 and KCa3.1 were detected in FRT-KCa3.1 cells. Lane 1: FRT-KCa3.1 UT, Lane 2: FRT-KCa3.1-SC, and Lane 3: FRT-KCa3.1-siRNA. GAPDH was used as a loading control. **(B)** SNAP-23 expression in FRT-KCa3.1-siRNA cells was reduced by 77 ± 5% (*n* = 6, *p ≤ 0.05) with respect to FRT-KCa3.1-SC cells. **(C)** In the presence of knockdown of SNAP-23, the KCa3.1 membrane expression in FRT-KCa3.1-siRNA cells was reduced by 56 ± 7% (*n* = 6, **p ≤ 0.01) with respect to FRT-KCa3.1-SC cells. **(D)** Ussing chamber experiment of FRT-KCa3.1 cells with SNAP-23 knock down. Representative traces of control FRT-KCa3.1 (black line), FRT-KCa3.1-SC control (dashed line) and FRT-KCa3.1-siRNA cells (dotted line) are shown. 1-EBIO promoted a significantly reduced K^+^ current in the FRT-KCa3.1-siRNA cells compared to control FRT-KCa3.1 UT cells or the FRT-KCa3.1-SC control cells. This current was abated by clotrimazole (10 µM). **(E)** Bar graph of K^+^ current of control FRT-KCa3.1, FRT-KCa3.1-SC and FRT-KCa3.1-siRNA cells treated with 1-EBIO. FRT-KCa3.1-siRNA cells demonstrated a significantly reduced current by 80 ± 11% compared to FRT-KCa3.1-SC cells (*n* = 6, *p ≤ 0.05).

SNAP-23 is one SNARE protein that assists the docking and incorporation of cargo vesicles arriving at the plasma membrane. Low and colleagues (1998) demonstrated that SNAP-23 was critical for the fusion of vesicles at both the apical and basolateral membranes of epithelial cells. Indeed, Leung et al. (1998) reported that SNAP-23 was required for the fusion of recycling endosomes to the BLM of MDCK cells. Also, Bertuccio et al. (2018) recently demonstrated SNAP-23 was required for insertion of KCa2.3 into the plasma membrane of HeLa cells. Even though KCa2.3 and KCa3.1 are in the same gene family there are many aspects of channel activation, inhibition and trafficking that differs between these channels (Gerlach et al., 2000, 2001; Wulff et al., 2000; Hamilton et al., 2003). Therefore, we investigated the role of SNAP-23 in the targeting of KCa3.1-containing vesicles to the BLM. As shown in Figure 3, specific siRNA for SNAP-23 reduced expression of SNAP-23 (FRT-KCa3.1-siRNA cells) by 77 ± 5% compared with FRT-KCa3.1-SC cells (Figure 3A upper panel, lanes 2 and 3; Figure 3B, dark gray bar versus light gray bar, *p* ≤ 0.05, n = 6). Correspondingly, the basolateral membrane expression of FRT-KCa3.1 was reduced by 56 ± 7% in the presence of SNAP-23 siRNA when compared with FTR-KCa3.1-SC cells (Figure 3A, middle panel lanes 2 and 3; Figure 3C, dark gray versus light gray bar, *p* < 0.05, *n* = 6). These data suggest there was reduced expression of KCa3.1 channels at the BLM with knockdown of SNAP-23.

Thus, we examined the functional expression of KCa3. l in the presence of knockdown of SNAP-23 with Ussing chamber experiments. As can be seen in Figures 3D,E, in the presence of reduced SNAP-23, the K^+^ current of the FRT-KCa3.1-siRNA cells (Figure 3D, dotted line, Figure 3E dark grey bar) was reduced by 80 ± 11% when compared with FRT-KCa3.1-SC cells (Figure 3D, dashed line, Figure 3E, light grey bar) (*p* ≤ 0.05, *n* = 6). These data demonstrate that SNAP-23 is crucial in the fusion of KCa3.1-containing vesicles with the BLM of epithelial cells.

### Role of Syntaxin 4 in the Targeting of KCa3.1-Containing Vesicles to the Basolateral Membrane

**FIGURE 4:**
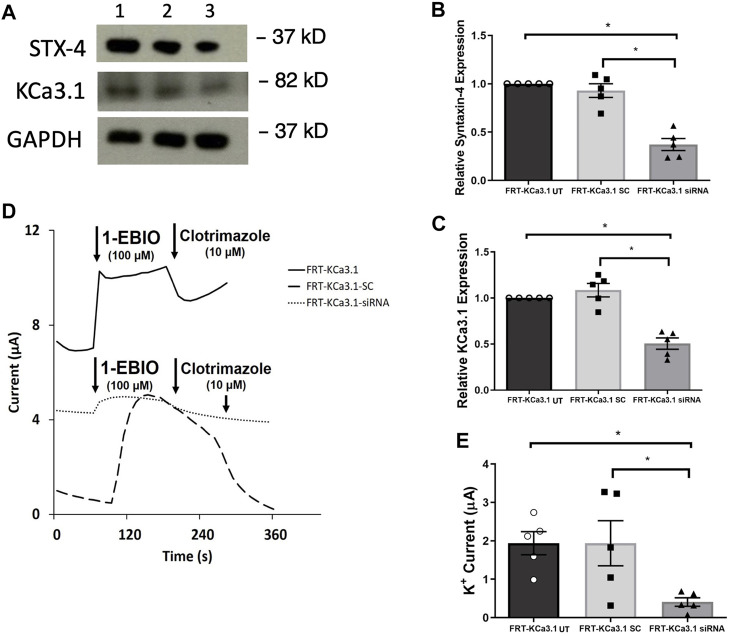
The membrane expression and function of KCa3.1 in response to the siRNA knockdown of STX-4. FRT-KCa3.1-BLAP cells were reverse transfected (20 pM, 48 h) with a scrambled siRNA (FRT-KCa3.1-SC) or siRNA to knock down STX-4 (FRT-KCa3.1-siRNA). **(A)** FRT-KCa3.1 UT (untransfected with any siRNA, UT), FRT-KCa3.1-SC, and FRT-KCa3.1-siRNA cells were run by immunoblot (8% gel). Lanes were loaded with 30 μg of protein. STX-4 and KCa3.1 were detected in FRT-KCa3.1 cells. Lane 1: FRT-KCa3.1, Lane 2: FRT-KCa3.1-SC, and Lane 3: FRT-KCa3.1-siRNA. GAPDH was used as a loading control. **(B)** STX-4 expression in FRT-KCa3.1-siRNA cells was reduced by 60 ± 6% (*n* = 5, *p ≤ 0.05) with respect to FRT-KCa3.1-SC cells. **(C)** In the presence of knockdown of STX-4, the KCa3.1 membrane expression in FRT-KCa3.1-siRNA cells was reduced by 54 ± 6% (*n* = 5, *p ≤ 0.05) with respect to FRT-KCa3.1-SC cells. **(D)** Ussing chamber experiment of FRT-KCa3.1 cells with STX-4 knock down. Representative traces of control FRT-KCa3.1 (black line), FRT-KCa3.1-SC cells (dashed line) and FRT-KCa3.1-siRNA cells (dotted line) are shown. 1-EBIO promoted a significantly reduced K^+^ current in the FRT-KCa3.1-siRNA cells compared to control FRT-KCa3.1 or the FRT-KCa3.1-SC control cells. This current was abated by clotrimazole (10 µM). **(E)** Bar graph of K^+^ current of control FRT-KCa3.1 UT, FRT-KCa3.1-SC control and FRT-KCa3.1-siRNA cells treated with 1-EBIO. FRT-KCa3.1-siRNA cells demonstrated a significantly reduced current by 78 ± 11% (*n* = 5, *p ≤ 0.05) compared to FRT-KCa3.1-SC cells.

STX-4 is another member of the SNARE family and plays a role in vesicle fusion machinery. Mostov and coworkers (Low et al., 1996) provided evidence that STX-4 was localized exclusively to the BLM of MDCK epithelial cells and was a target SNARE for transport vesicles. Additionally, Weimbs and colleagues (Low et al., 2006) reported that STX-4 formed clusters with other proteins during fusion of transport vesicles and they demonstrated that STX-4 co-localized with SNAP-23. Recently, Bertuccio et al. (2018) reported that STX-4 was important in the trafficking of KCa2.3 to the plasma membrane of HeLa and brain microvascular endothelial cells (hCMEC). Based on those findings, we examined whether STX-4 was crucial for the targeting of KCa3.1-containing vesicles to the BLM of our FRT-KCa3.1 cells. Knockdown of STX-4 reduced cellular STX-4 of FRT-KCa3.1-siRNA cells by 60 ± 6% compared with control FRT-KCa3.1-SC cells (Figure 4A upper panel, lanes 2 and 3; Figure 4B, dark gray bar versus light gray bar, *p* ≤ 0.01, n = 5). Similarly, with the knockdown of STX-4, the basolateral membrane expression of KCa3.1 was decreased by 54 ± 6% compared with FTR-KCa3.1-SC cells (Figure 4A, middle panel lanes 2 and 3; Figure 4C, dark gray bar versus light gray bar, *p* < 0.05, *n* = 5).

Finally, to determine the effect of reduced STX-4 on the functional expression of KCa3.1, we assessed the K^+^ current in the presence or absence of knockdown of STX-4. As shown in Figures 4D,E, with reduced STX-4, the K^+^ current of the FRT-KCa3.1-siRNA cells (Figure 4D, dotted line, Figure 4E dark gray bar) was reduced by 78 ± 11% when compared with control FRT-KCa3.1-SC cells (Figure 4D, dash line, Figure 4E, light gray bar) (*p* ≤ 0.05, *n* = 5). These data demonstrate STX-4 is essential for the delivery of KCa3.1 to the BLM of epithelial cells.

### Protein-Protein Associations Between KCa3.1, VAMP3, SNAP-23 and STX-4

**FIGURE 5:**
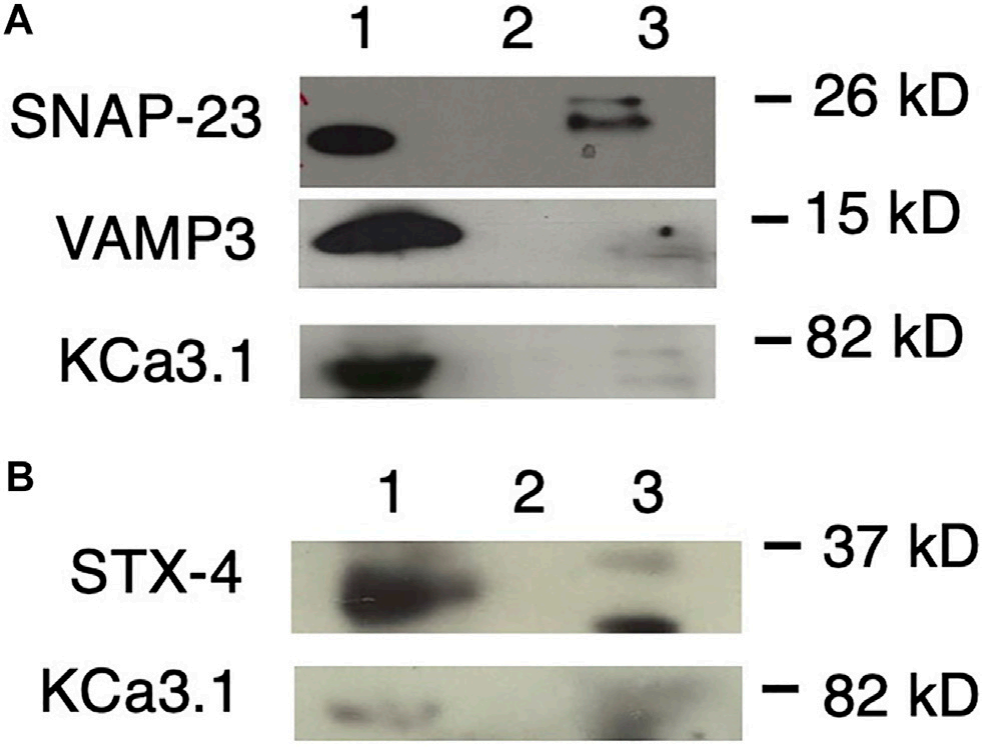
KCa3.1 co-immunoprecipitates (Co-IP) with VAMP3, SNAP-23 and Syntaxin-4 (STX-4). KCa3.1-BLAP in FRT cells was used as bait to determine protein associations with the SNARE proteins VAMP3, SNAP-23 and STX-4. **(A)** Upper Panel: The blot was probed with anti-SNAP-23 antibody. Lane 1 - SNAP-23 lysate control; Lane 2 - IgG negative control; and Lane 3—pulled down of SNAP-23 (*n* = 5). Middle Panel: The blot was stripped and reprobed with anti-VAMP3 antibody. Lane 1—VAMP3 lysate control; Lane 2 - IgG negative control; and Lane 3—VAMP3 pulled down by Co-IP (*n* = 6). Lower Panel: The blot was stripped and reprobed with anti-streptavidin antibody. Lane 1—KCa3.1 lysate control; Lane 2—IgG negative control; and Lane 3—KCa3.1 pulled down by Co-IP (*n* = 6). **(B)** Upper Panel: The blot was probed with anti-STX-4 antibody. Lane 1—STX-4 lysate control; Lane 2—IgG negative control; and Lane 3—STX-4 pulled down by Co-IP (band is running high, *n* = 6). Lower Panel: The blot was stripped and reprobed with anti-streptavidin antibody to detect KCa3.1. The KCa3.1 band is diffuse likely due the membrane being stripped. Lane 1—KCa3.1 lysate control; Lane 2—IgG negative control; Lane 3—KCa3.1 pulled down by Co-IP (*n* = 6). These data suggest protein associations occur between KCa3.1 and VAMP3, SNAP-23 and Syntaxin-4.

Anterograde trafficking of proteins to a specific cell’s membrane is a complex process. The membrane location of an ion channel is dependent upon the trafficking of the ion channel -containing vesicle, and then, tethering, fusion and incorporation of the ion-carrying vesicle with the plasma membrane. The dynamics of vesicle fusion at the plasma membrane has been a subject of much investigation and is achieved by protein-protein associations of vesicle-surface proteins with several SNARE proteins (Carmosino et al., 2010). Recently, Bertuccio et al. (2018) reported that KCa2.3 co-immunoprecipitated (Co-IPed) with both SNAP-23 and STX-4 in Human embryonic kidney epithelial cells and hCMEC endothelial cells. Their data suggested that KCa2.3 and those SNARE proteins are present within a protein complex critically important in the incorporation of KCa2.3-containing vesicles into the plasma membrane. Using our FRT-KCa3.1-BLAP cell line, we performed Co-IP experiments to determine whether KCa3.1 associates with SNAP-23, VAMP-3 and/or STX-4 - suggesting these proteins might exist as a protein complex. Therefore, KCa3.1 was used as bait for the Co-IP experiments. Briefly using KCa3.1 as ‘bait’, KCa3.1 was labelled with streptavidin as described above. Once FRT cells were harvested, the FRT cells were incubated with anti-streptavidin antibody for KCa3.1 followed by IB for the appropriate endogenously expressed SNARE proteins or KCa3.1 (Figure 5).

As shown in Figure 5A, with using KCa3.1 as bait, and probing with α-SNAP-23 antibody, SNAP-23 was Co-IPed by KCa3.1 (upper panel, Co-IP is Lane 3 pull down of SNAP-23, lane 1 is lysate; *n* = 5). After stripping the same membrane and re-probing with α-VAMP3 antibody, KCa3.1 had pulled down VAMP3 (middle panel, Co-IP is Lane 3 pull down of VAMP3, lane 1 is lysate; *n* = 6). Finally, the membrane was stripped, again, and re-probed with α-streptavidin antibody. KCa3.1 (control) is shown in the lower panel (lane 3 pull down of KCa3.1, lane 1 is lysate; *n* = 6, Figure 5A). These results confirm, for the first time, potential associations between KCa3.1, SNAP-23 and VAMP-3. Therefore, KCa3.1, SNAP23 and VAMP3 appear to be members of the same protein complex during the incorporation of the KCa3.1-containing vesicles into the BLM.

Given that Bertuccio et al. (2018) demonstrated that STX-4 was Co-IPed by KCa2.3, and was critical in the trafficking of KCa2.3, we determined whether KCa3.1 associated with STX-4. As seen in Figure 5B, when probing with α-STX-4 antibody, STX-4 was Co-IPed by KCa3.1 (upper panel, Co-IP is lane 3 pull down of STX-4, lane 1 is lysate; *n* = 6). After stripping the membrane and re-probing with α-streptavidin antibody; KCa3.1 (control) is shown in the lower panel (Co-IP Lane 3, lane 1 is lysate; *n* = 6, Figure 5B). Here, we provide the first evidence that KCa3.1 associates with STX-4.

Overall, our Co-IP experiments suggest that KCa3.1 associates with VAMP3, SNAP-23 and STX-4. These data indicate that these proteins most likely exist in the same stable protein complex at the BLM resulting in the incorporation of KCa3.1-containing vesicles into the BLM of epithelial cells.

## Discussion

The overall trafficking of KCa3.1 to the plasma membrane and its subsequent endocytosis is an evolving story. The retrograde trafficking of KCa3.1 is well established. Balut et al. (2010b) reported that once in the plasma membrane, KCa3.1 is endocytosed within 90 min by an ubiquitylation-dependent mechanism. Following endocytosis, KCa3.1 is then targeted for degradation by lysosomes thru a Rab7-and multivesicular/ESCRT-dependent pathway (Balut et al. (2011). Additionally, KCa3.1 is not deubiquitylated at the BLM, but must occur further downstream in the degradation pathway (Lee et al., 2017). To maintain an adequate number of channels at the membrane requires regulating the rates of retrograde and anterograde trafficking of the channel. However, our understanding of the anterograde trafficking of KCa3.1 is still emerging.

We have reported that the anterograde trafficking of KCa3.1 is dependent upon Rab-1, Rab-8, but not dependent upon transferrin- nor RME-1 (Receptor-mediated endocytosis 1) -positive recycling endosomes, nor AP-1 adaptor protein µ1B, although dependent upon the cytoskeleton and myosin-Vc (Bertuccio et al., 2014; Farquhar et al., 2017). The above experiments have provided a mechanistic understanding of the anterograde trafficking of KCa3.1 from the ER to the BLM (further reviewed in Devor et al., 2020). Nonetheless, the molecular mechanism responsible for the incorporation of KCa3.1-containing vesicles into the BLM is still undetermined. Therefore, we asked the question which SNARE proteins were key for the incorporation of KCa3.1-containing vesicles into the BLM of epithelial cells?

Here, we used a siRNA knock-down approach to reduce endogenous SNARE proteins coupled with western blot experiments to examine the cytosolic levels of the SNAREs and the membrane expression of KCa3.1. As seen in Figures 2A–4A,B, siRNA knockdown of VAMP3, SNAP-23 or STX-4 significantly reduced the cytosol levels of the SNARE proteins resulting in decreased membrane expression of KCa3.1 in the FRT cells (Figures 2A–4A,C). Using Ussing chamber experiments, we demonstrated reduced functional expression of KCa3.1 at the BLM in the presence of SNARE knockdown (Figures 2D–4D,E). Finally, using Co-IP experiments, we identified protein associations between KCa3.1 (bait) and VAMP3, SNAP-23 and STX-4 (Figure 5). These results provide the first evidence that KCa3.1 is incorporated into the BLM of epithelial cells via a VAMP3-, SNAP-23- and STX-4-dependent mechanism. Also, this is the first report of VAMP3 participating in any cargo-carrying vesicle incorporation mechanism for any member of the KCNN gene family. Finally, our results provide the first evidence that KCa3.1 and these SNARE proteins may participate in a stable protein complex during the incorporation of KCa3.1-containing vesicles into the BLM.

The v-SNARE VAMP3 is a key SNARE protein residing in the membrane of cargo-containing vesicles being targeted to the cell surface (Ravichandran et al., 1996; Veale et al., 2010) and VAMP3 is involved in the incorporation of cargo-containing vesicles trafficking via the cytoskeletal elements to the BLM (Fields et al., 2007). Fields et al. (2007) demonstrated that VAMP3 and STX-4 formed SNARE-pairs during the trafficking of AP-1B-dependent cargo vesicles to the BLM of MDCK cells. We have reported that the cytoskeleton is crucial in the trafficking of KCa3.1 to the BLM (Farquhar et al., 2017). Additionally, Bertuccio et al. (2014) demonstrated that Rab8 is pivotal in trafficking KCa3.1. So, it is plausible that Rab8 aids in the recruitment of VAMP3 at the level of the cytoskeleton along the trafficking pathway of KCa3.1-containing vesicles (Farquhar et al., 2017). Indeed, Finetti et al. (2015) reported that Rab8 acted similar to a regulator of T-cell receptor recycling in Jurkat cells by enlisting VAMP3 in the immune synapse. These authors noted that the localization of VAMP3 was impaired in T-cells expressing DN-Rab8. Interestingly, Nicolaou et al. (2007) demonstrated KCa3.1 was evenly distributed on the plasma membrane of non-activated T cells, but that upon antigen presentation, KCa3.1 was targeted to the immunological synapse with F-actin. This highlights the possibility that the combination of Rab-8 and VAMP3 likely play a critical role in the targeting of KCa3.1 to the plasma membrane in a host of cellular systems.

SNAP-23 and STX-4 play major roles in the protein machinery involved in delivering cargo-carrying vesicles to the BLM in epithelial cells. Indeed, Weimbs and colleagues (Low et al., 2006), using immunostaining in MDCK cells, demonstrated that SNAP-23 colocalized with STX-4 clusters and SNAP-23 clusters overlapped with STX-4. These authors reported a significant fraction of STX-4 clusters also contained SNAP-23, indicating that these proteins were potential functional participants of the vesicle fusion machinery (Low et al., 2006). Also, Ravichandran et al. (1996) reported that STX-4 and SNAP-23 bound together determined by *in vitro* binding assays. Likewise, Predescu et al. (2005) used high-resolution fluorescence microscopy and gold labelling electron microscopy and demonstrated that SNAP-23 and STX-4 formed cholesterol-dependent clusters in specific areas which regulated caveolar fusion in the plasma membrane in human lung microvascular endothelial cells. With a different approach than Co-IP experiments, Bertuccio et al. (2018) used ‘membrane footprints’ of hCMEC/D3 and demonstrated close associations of KCa2.3 with both STX-4 and SNAP23 in caveolin-1-rich domains in the plasma membrane. Here, we report associations between KCa3.1 and SNAP-23 (Figure 5A) and between KCa3.1 and STX-4 (Figure 5B). Furthermore, we demonstrated that siRNA knockdown of either cellular SNAP-23 or STX-4 resulted in reduced surface expression and functional expression of KCa3.1 (Figures 3, 4). We are confident that SNAP-23 and STX-4 are significant players in the fusion machinery of KCa3.1 containing vesicles trafficked to the BLM of epithelial cells.

We have identified the SNARE proteins crucial in the final stages of incorporating KCa3.1-containing vesicles in the BLM. Although, prior to the role of SNARE proteins, these vesicles must be tethered to the BLM which is accomplished by protein complexes. The Exocyst complex is an octomeric protein complex important for tethering cargo-containing vesicles to the plasma membrane (Yeaman et al., 2001; Hsu et al., 2004; Nishida-Fukuda, 2019). Therefore, what is still yet to be determined is (are) the molecular player(s) involved in the tethering of KCa3.1-containing vesicles to the BLM. The role of the Exocyst in the trafficking of KCa3.1 is an unexplored avenue of research, and thus, warrants further investigation.

**FIGURE 6:**
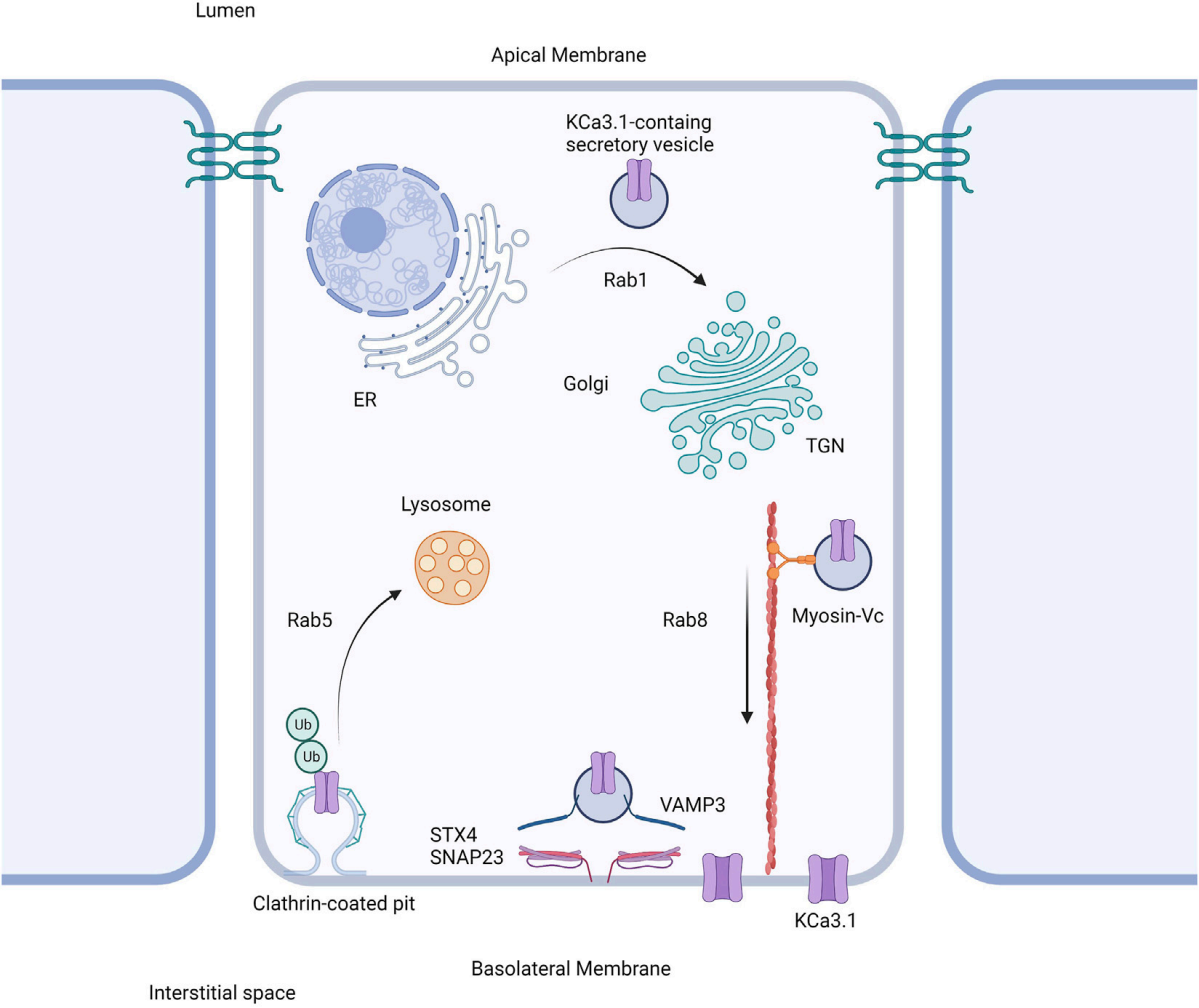
Proposed cell model of the role of SNARE proteins in the targeting of KCa3.1 to the basolateral membrane. After being trafficked to the basolateral membrane in a Rab1, Rab8, and myosin-Vc dependent pathway, the SNARE proteins VAMP3, SNAP-23, and STX4 form a protein complex resulting in the incorporation and fusion of the KCa3.1-containing vesicle with the basolateral membrane. Thereupon KCa3.1 is inserted into the basolateral membrane. Figure created with BioRender. ER, endoplasmic reticulum; TGN, *trans*-Golgi Network; Ub, ubiquitin.

Based on our current study together with our previously published work, we propose the following model shown in Figure 6. The trafficking of KCa3.1-containing vesicles from the ER to the Golgi apparatus is a Rab1-dependent process (Bertuccio et al., 2014). Additionally, trafficking of the vesicles from the Golgi to the BLM is dependent upon Rab8, Myosin Vc, and the microtubule and microfilament cytoskeleton (Bertuccio et al., 2014; Farquhar et al., 2017). Lastly, the fusion and incorporation of KCa3.1-containing vesicles with the BLM requires VAMP3, SNAP-23 and STX-4. The only link in the trafficking of KCa3.1 to the BLM, which remains to be investigated is the tethering mechanism.

In summary, we provide the first evidence that the SNARE proteins VAMP3, SNAP-23, STX-4 are critical for the incorporation of KCa3.1 channels into the BLM of polarized epithelial cells. Also, this is the first report of the participation of VAMP3 in the trafficking of any member of the KCNN gene family. Herein, using Co-IP experiments, we demonstrate that KCa3.1 associates with VAMP3, SNAP-23 and STX-4. Whether these proteins form a stable quaternary macro-molecular protein complex during the incorporation of KCa3.1-containing vesicles into the BLM is unknown. However, our re-probing of Co-IP blots with different antibodies are suggestive, at least, for binary protein associations. These combined data suggest that KCa3.1, VAMP3, SNAP-23, and STX-4 likely exist in the same stable protein complex during the incorporation of KCa3.1-containing vesicles into the BLM of epithelial cells.

## Data Availability

The raw data supporting the conclusions of this article will be made available by the authors, without undue reservation.
