# Supplementary figures and images for: Role of SNARE Proteins in the Insertion of KCa3.1 in the Plasma Membrane of a Polarized Epithelium
Source: Front Physiol. 2022 Jun 27;13:905834. doi: 10.3389/fphys.2022.905834 (PMC9271999; doi:10.3389/fphys.2022.905834)

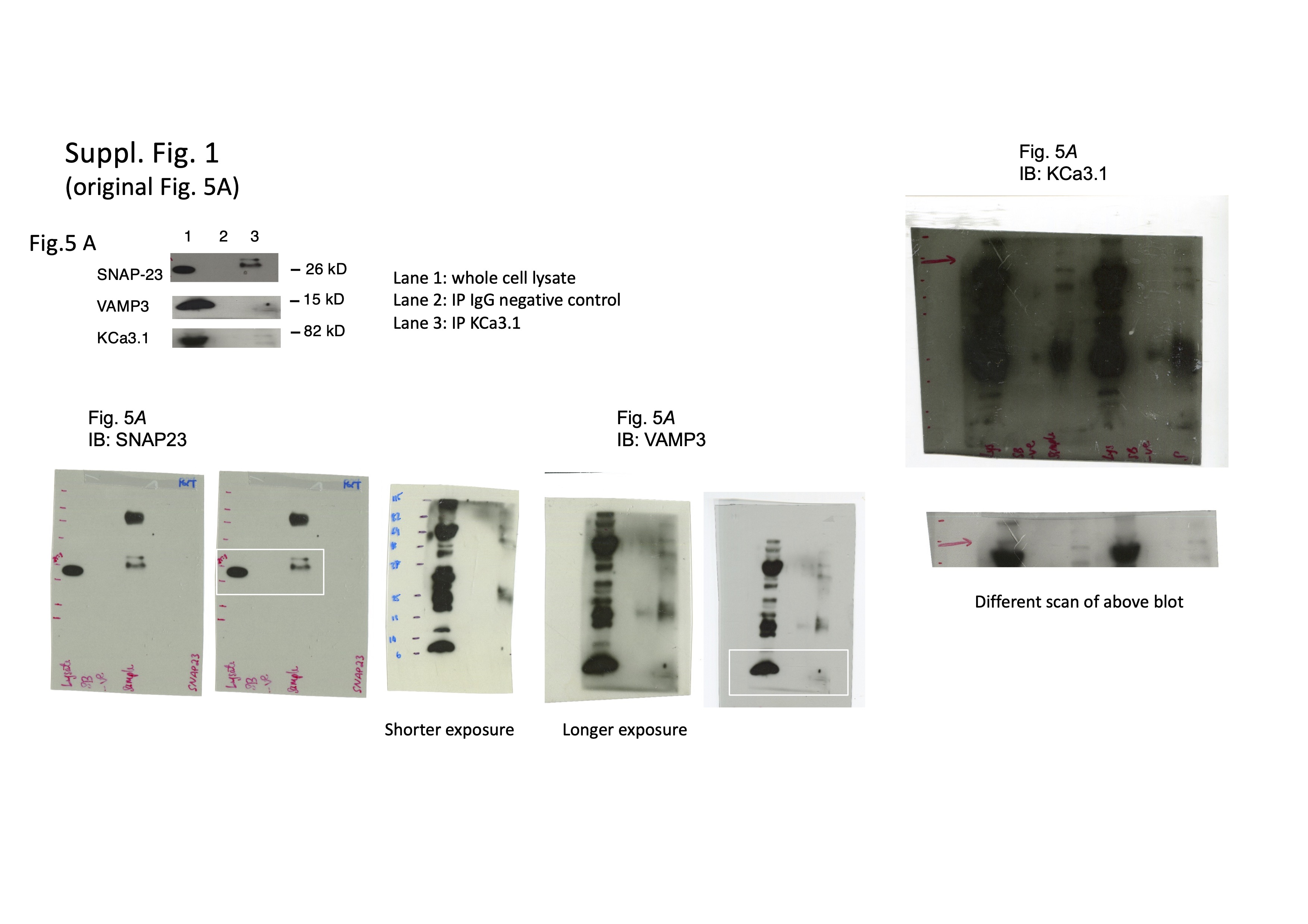

Supplement: Supplementary file 1 [file Image1.JPEG]

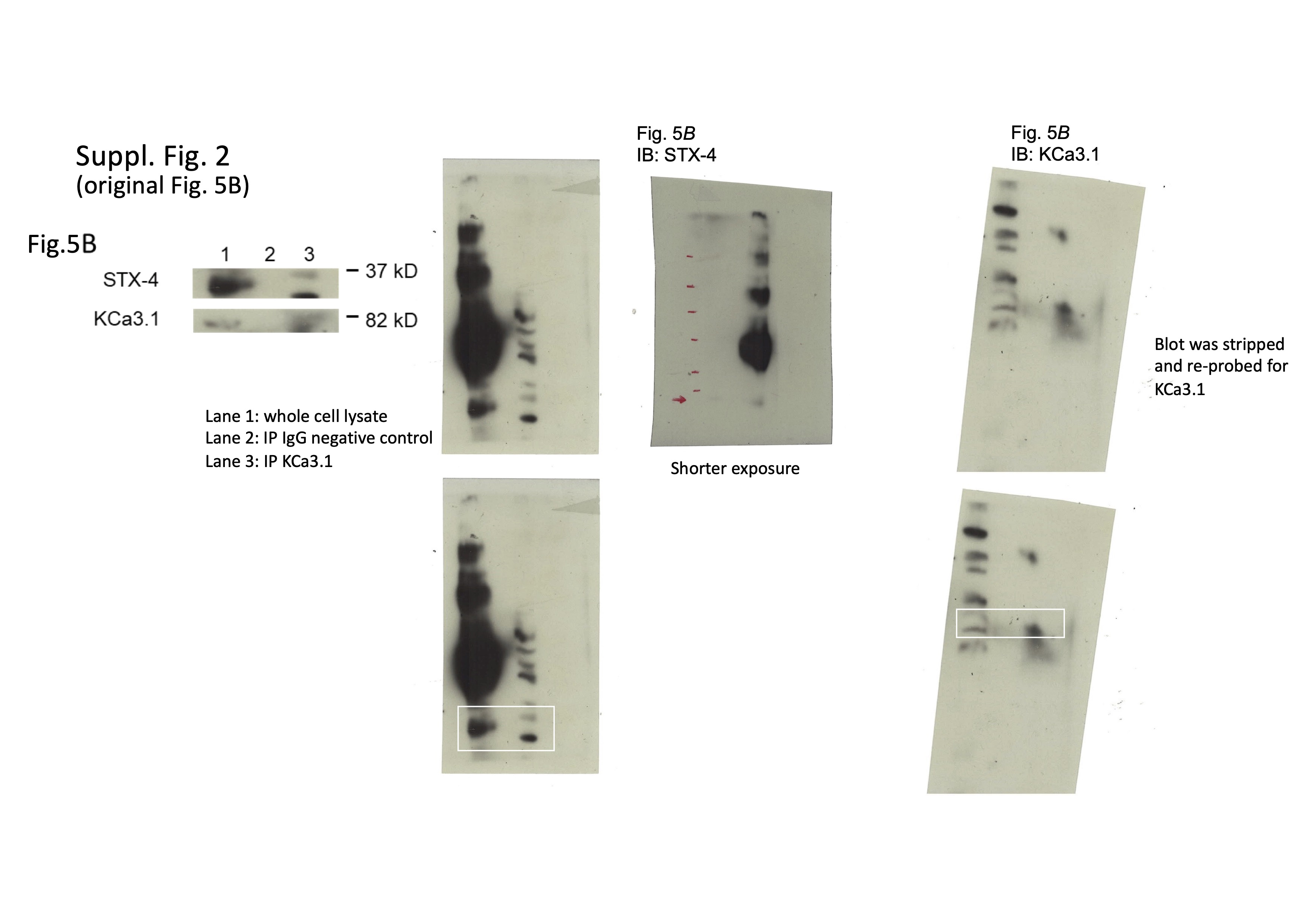

Supplement: Supplementary file 2 [file Image2.JPEG]
